# Emerin acts as a mechanosensor linking force transmission and disease

**DOI:** 10.3389/fonc.2026.1882684

**Published:** 2026-07-30

**Authors:** Emily Hansen, James M. Holaska

**Affiliations:** 1 Department of Biomedical Sciences, Cooper Medical School of Rowan University, Camden, NJ, United States; 2 Cell and Molecular Biology Program, Rowan-Virtua School of Translational Biomedical Engineering and Sciences, Stratford, NJ, United States

**Keywords:** force transmission, LINC, mechanotransduction, metastasis, tumor microenvironment (TME), emerin, cancer

## Abstract

For cells to respond to mechanical and biochemical cues in their environment, signals from the extracellular environment must be transmitted across the plasma membrane and into the nucleus to create the correct physical, transcriptional, and biochemical responses. This process is called mechanotransduction, which often becomes dysfunctional during cancer transformation to promote uncontrolled growth and metastatic progression. Emerin, an inner nuclear membrane (INM) protein that contributes to maintaining nuclear architecture, can receive these extracellular and extranuclear signals directly through the Linker of the Nucleoskeleton and Cytoskeleton (LINC) complex. Through association of LINC with the cytoskeleton and plasma membrane, it directly transmits force from the plasma membrane to the nucleus. Emerin has also been implicated in regulating biochemical mechanotransduction pathways, such as YAP/TAZ and MKL1/MRTFA, where emerin integrates mechanical signals with transcriptional responses. This review will discuss nuclear mechanotransduction and emerin’s role as a central node that integrates mechanical signals to regulate cellular responses within complex extracellular environments, and how dysfunction contributes to cancer progression.

## 1 Introduction

### 1.1 Mechanotransduction

Cellular function and survival depend on the ability of the cell to sense and respond to changes in the extracellular environment. Historically, biologists explored these concepts by modulating environmental conditions and observing both organismal and cellular responses. With the invention of microscopy, these responses could be observed and recorded at subcellular levels, revealing that intracellular structures can and do respond and adapt to extracellular cues. A foundational study in 1954 by Hugh and Andrew Huxley established key principles in mechanobiology through studies of actomyosin-based force transmission within muscle cells (1). That same year, Sanford, Likely, and Earle showed sarcoma cells could proliferate in soft agar independently of substrate attachment, unlike their non-transformed counterparts derived from the same clone (2), which highlighted an early hallmark of cancer and underscored the importance of cell adhesion in normal cellular function. Together, these observations provided evidence for cells responding biochemically to physical stimuli, laying the groundwork in the field of mechanobiology.

Since these early studies, the field of mechanobiology has evolved to include highly conserved tension-sensing pathways that convert mechanical stimuli from the extracellular environment into internal biochemical signals (3). This tension-sensing process transmits mechanical force from the extracellular matrix (ECM) to the nucleus through coordinated interactions between the cytoskeleton and nuclear envelope (NE).

Through these physical connections between the cell surface and the nucleus, cells are able to integrate extracellular mechanical cues into the activation and/or repression of downstream pathways to regulate cellular function, a process known as mechanotransduction (4, 5).

Mechanotransduction is critical in regulating physiological processes by enabling cells to both interpret *and* respond to cues in their environment (6). In the context of cancer and mechanobiology, it is the stiffness of the cellular microenvironment that often regulates cellular behavior (7). *Stiffness* describes the ability of a material to resist deformation upon applied force. Changes in ECM mechanics (i.e., stiffness) influence processes such as proliferation, differentiation, and migration (8). This is especially relevant for cancer transformation, as substrate stiffening in cancer cells has been associated with increased proliferation, increased migration and invasion, and ‘dedifferentiation.’ (9–12).

In cancer, tissue mechanics play a key role, where tumor stiffening is often used as a clinical marker (13). Changes in stiffness of a cell or tumor’s microenvironment are transduced, using both biochemically and biophysically based mechanisms, to potentiate changes in gene expression and cellular architecture (4, 14, 15). With overstimulation of mechanotransduction receptors and signaling pathways, or mutations in proteins in these pathways, mechanotransduction becomes overactivated or attenuated, contributing to the disease phenotypes. In the case of cancer, tumor cells can exploit mechanotransduction (or defects therein) to promote cancer progression and enable metastasis by altering their responses to the tumor microenvironment (TME) surrounding them to facilitate metastasis.

### 1.2 The TME and mechanotransduction

In addition to the accumulation of mutations that imbue cells with invasive properties, another important aspect of cancer progression is the TME. The TME is comprised of macromolecules that help provide biochemical and mechanical signals, nutrients, and scaffolding to the tumor (16). This TME consists of the cancer cells themselves, fibroblasts, immune cells, and signaling molecules that help potentiate cancer progression (17, 18). Stromal and immune cells within the TME regulate tumor metabolism and metastatic progression (19).

The ECM of the TME forms the basement membrane of the tumor. This is a major site of metastasis into the blood vessels, if the cells become more malleable to be able to fit through gaps in the basement membrane (20). The ECM itself is dynamic, responding to physiological conditions of the body. ECM stiffening regulates NE composition and chromatin accessibility within the nucleus through mechanotransduction to regulate both nuclear structure and gene expression (21–24). This altered mechanotransduction from the ECM and TME directly to the nucleus via the linker of the nucleoskeleton and cytoskeleton (LINC) complex has been implicated in cancer (25–29).

As tumors progress and cells proliferate, more ECM is produced, which is largely made of stiff collagen, further stiffening the TME and causing more dynamic changes in cellular functions, largely through mechanotransduction (30). Increased ECM stiffening promotes cell spreading, enhanced adhesion, contractility, and invasive behavior in cancer cells (31–34). One study reported increased stiffness further drives both cancerous and noncancerous mammary epithelial cells to a more malignant phenotype, even though they already exhibit increased mechanotransduction (31). In addition to collagen, fibronectin is an important ECM protein that connects collagen in the ECM to integrins in the plasma membrane to potentiate cell motility through cytoskeletal remodeling (35). As with other ECM proteins, fibronectin is reportedly upregulated in multiple cancers (32, 33, 36). Fibronectin is predicted to assist in angiogenesis (37), allowing for tumor growth and cancer progression (38). Proteoglycans within the ECM allow for cells to resist compressive forces and assist in migration (39). Depending on the study, proteoglycans can either promote or limit cancerous traits such as angiogenesis, proliferation, and the epithelial-mesenchymal transition (EMT) (40), garnering attention from the cancer field.

As tumors progress, the ECM also develops atypical compositions, which disrupt the function of the basement membrane, contributing to EMT (41, 42) and eventually metastasis. These abnormal processes are driven in part by ECM-associated signaling pathways that activate focal adhesion kinase, which promotes survival signaling (43). The increased deposition of collagen, which is a major constituent of the ECM (44), acts as a protective barrier in the body. Unfortunately, this also blocks the entry of cancer-targeted immune cells and cancer therapies (45). Measuring increased tumor stiffness serves as a useful tool for diagnosing and detecting cancers in a clinical setting because increased collagen deposition in the ECM is linked to metastatic progression (46).

Additionally, cancer-associated fibroblasts (CAFs) actively rearrange the ECM through the secretion of growth factors and enzymes, such as matrix metalloproteinases (MMPs), which promotes angiogenesis (47) through degrading ECM components. MMPs also cleave cell-cell adhesion proteins such as E-cadherin, which in turn further disrupts interactions between cells and promotes migration (47, 48). These MMPs can also break down collagen within the ECM to facilitate EMT and migration and invasion (41). CAFs can also secrete transforming growth factor beta (TGF-β), which creates a pro-tumorigenic and pro-EMT environment (49). These CAF-mediated ECM changes contribute significantly to modulating mechanotransduction (49). There are a number of MMP family members that are frequently upregulated in CAFs and tumor cells (50), including MMP-2, MMP-9, and MMP-14, all of which are involved in stimulating migration (51).

It is important to emphasize that as tumors progress, the continued and altered extracellular forces (e.g., TME stiffening) are likely to increase mechanotransduction to modulate pro-tumorigenic and -metastatic characteristics. This stiffening will continue to transmit force to cells, further triggering mechanotransduction events, leading to changes in nuclear structure and gene expression that impact a cell’s ability to migrate (52). For example, a recent study showed that highly invasive MDA-231 breast cancer cells migrated deeper into dense extracellular matrices than loose matrices, something that less invasive, poorly metastatic MCF7 cells were unable to do (53), although both are considered to be cancerous. Stiff matrices were also found to contribute to abnormal mammary epithelial tissue behavior of mammary epithelial cells when compared to cells on softer matrices; these cells exhibited greater spreading, adherence, and response to epidermal growth factor (EGF) *in vitro* (31). Modulating ECM crosslinking *in vivo* was able to alter tumor formation (32), demonstrating the importance of the ECM architecture in cancer progression.

Collectively, these findings support a model in which changes in the TME dysregulate mechanotransduction in cancer cells, serving as a feed-forward mechanism during cancer progression. These forces are transmitted to the nucleus, either directly or indirectly, where NE proteins integrate mechanical signals to regulate a biochemical response. There are several reviews that elegantly describe mechanotransduction in this way (3, 4, 7, 21, 54, 55); however, less attention has been given to *how* these signals are integrated by the INM proteins and *how* these signals become downstream cellular responses. Emerin is a unique protein in that it not only directly receives signals from the LINC complex (56–58), but it directly interacts with the nuclear lamina (57–60), chromatin regulation machinery (24, 61, 62), and mechanosensitive transcription (63–66). This review focuses on emerin as a mechanosensor that links physical stimuli to changes in nuclear architecture, chromatin organization, and gene expression and how disruptions in these functions contributes to cancer progression.

## 2 Mechanotransduction

Cells first sense mechanical cues from the ECM at the plasma membrane through integrins, transmembrane receptors that bind ECM proteins such as collagen and fibronectin through their extracellular domains (67). Integrins’ cytoplasmic tails recruit focal adhesion proteins such as talin, vinculin, and focal adhesion kinase (FAK), which are collectively referred to as focal adhesions (68, 69). Focal adhesions physically couple the ECM to the cytoskeleton (67), enabling force transmission and the activation of mechanotransduction pathways.

### 2.1 The LINC complex

**Figure 1:**
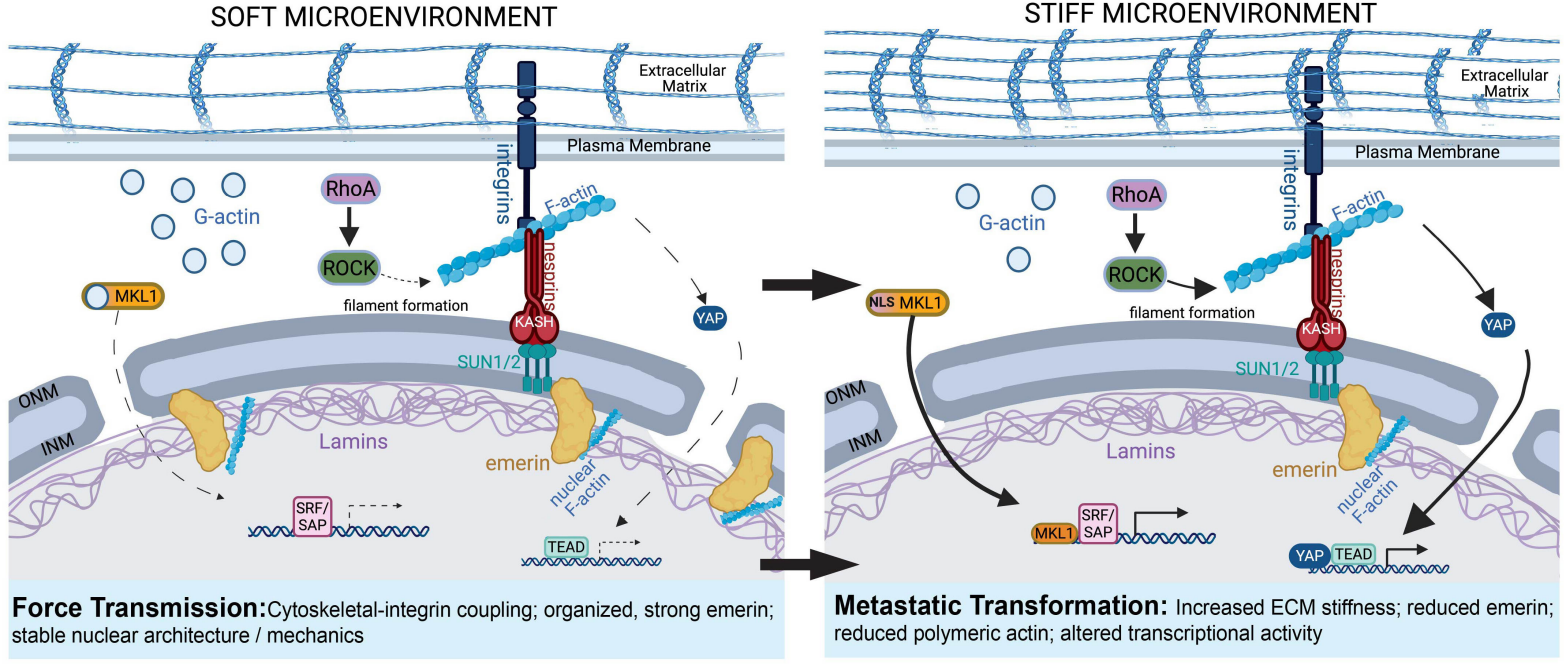
Proposed mechanism of mechanotransduction. Mechanical signals from the ECM are transmitted into the cell through integrin-based adhesions at the plasma membrane which connect to the cytoskeleton. These forces are transmitted intracellularly to the nucleus through the LINC complex, comprised of nesprin proteins at the ONM and SUN proteins in the INM. Cytoskeletal tension, regulated in part by RhoA/ROCK signaling, increases actin polymerization and contractility to transmit force. These mechanical inputs influence transcriptional pathways through the increased F-actin and decreased G-actin (blue circles), which causes increased nuclear localization of MKL1 and YAP. In the nucleus, MKL1 and YAP activate transcription by binding to coactivators SRF/SAP and TEAD family members, respectively. LINC signaling through emerin in response to substrate stiffening also results in less emerin expression, which weakens nuclear structure by removing a key ‘strut’ linking lamins and nuclear F-actin to the INM. The result of these nuclear structural and transcriptional changes drives cancer transformation. Created with https://www.BioRender.com.

Force is transmitted directly from the plasma membrane to the nucleus through the LINC complex. The LINC complex forms a continuous connection between the cytoskeleton and the nuclear interior, linking adhesion proteins at the plasma membrane to the NE via microtubules, actin filaments, and intermediate filaments (68, 70, 71). The LINC complex is composed of large, spectrin-repeat proteins named nesprins, which are bound to the cytoskeleton via their N-terminus and are embedded in the NE via their C-terminus (72–74). Nesprins use their C-terminal KASH domain to interact with the C-terminus of SUN-domain proteins in the lumen of the NE (75, 76). SUN proteins are INM proteins that are positioned such that the N-terminus is located in the nucleoplasm and the long C-terminus is located in the lumen of the NE. SUN proteins form a homotrimer that binds to three KASH proteins; this interaction is required for nesprin localization to the NE (70, 77). The SUN proteins function to transmit signals from nesprins into the nucleus through other INM proteins, such as emerin, to initiate a response to these mechanical stimuli (**Figure 1**) (75, 76). Disruption of the LINC complex blocks transmission of mechanical signals to the nucleus (78), highlighting the necessity for the LINC complex in mechanotransduction.

Though mechanotransduction is often referred to as the transmission of forces from the ECM to the nucleus, there is also evidence that LINC transmission is bidirectional (79). Infante et al., describe a “digest-on-demand” strategy which relies on an active LINC complex to alter endosome polarization and collagenolysis for migration through constrictive environments and tumor cell invasion/metastasis (80). Another report states that SUN2 LINC complexes signal from the NE *through* the cytoplasm to regulate focal adhesions and RhoA activity in HeLa cells (81). Similarly, in human myoblasts, it is reported that the nuclear-cytoskeletal linkage is important in tuning cellular stiffness to match their environments through its inside-out connection with focal adhesions and other related proteins (82). Disruption of the LINC complex has also been reported to impair the organization of the actin cytoskeleton and cytoskeletal dynamics (83), supporting the importance for further research in bidirectional mechanotransduction.

### 2.2 MKL1 signaling

Mechanotransduction-stimulated changes in actin dynamics can regulate the activity of mechanosensitive transcription factors, such as pro-oncogenic megakaryoblastic leukemia protein-1 (MKL1) (84–86). Inactive MKL1 is in the cytoplasm bound to G-actin. Upon actin polymerization, which occurs upon mechanical stimulation through focal adhesions, cytoplasmic G-actin levels decrease, causing MKL1 to be released from G-actin, thereby exposing a nuclear localization signal and entry of MKL1 into the nucleus to activate transcription with its co-regulators SRF (Serum Response Factor) and SRF-associated protein (SAP) (87). Therefore, actin polymerization allows more nuclear MKL1 to activate transcription of genes, many of which specifically affect migration and invasion (88). For example, many of these SRF- and SAP-dependent genes increase actin and cytoskeletal function (85, 87).

Although MKL1 activation is tightly linked to F-actin (85), the upstream regulator of cytoskeletal tension that largely drives these actin dynamics is controlled by RhoA/ROCK signaling (89). RhoA/ROCK (Rho-associated protein kinase) signaling is important for mechanotransduction through its regulation of actin dynamics. When activated in response to extracellular stimuli, RhoA GTPase activates ROCK to increase actin polymerization (89). Active ROCK phosphorylates LIM Kinase, which inactivates the depolymerization protein cofilin (90). This increased F-actin formation results in activation of MKL1 (63, 85). In addition to the RhoA/ROCK signaling pathway increasing the formation and elongation of F-actin polymers, it also increases focal adhesion formation, signaling, and myosin phosphorylation (89). This pathway ultimately increases cell contractility and overall cell stiffness. As RhoA/ROCK responds to extracellular signals, it serves as a major signaling node of mechanotransduction in parallel with MKL1 activation. Through this contractility, RhoA/ROCK signaling increases the force transmission across the integrins, focal adhesions, and cytoskeleton to the LINC complex to alter mechanosensitive gene expression and resulting nuclear architecture (91). This activation enhances actin polymerization and cytoskeleton tension that can be sensed by the nucleus (63, 85, 92).

### 2.3 YAP/TAZ signaling

In addition to MKL1 signaling, ECM stiffening and cytoskeletal tension also regulate the mechanosensitive effectors YAP (Yes-associated protein) and TAZ (Transcriptional coactivator with PDZ-binding motif). YAP and TAZ remain phosphorylated by LATS1/2 (Large tumor suppressor kinases 1/2) and inactive in the cytoplasm until mechanical stimulation, such as cell stretching or changes in ECM stiffening (93–95). Merlin (also known as neurofibromin type 2) is an upstream effector of Hippo and is responsible for regulating the activity of LATS1/2 (96). Upon mechanical stimulation, integrin-linked kinase (ILK) is recruited, inactivating merlin through ILK-assisted phosphorylation (97–99). Merlin inactivation inactivates LATS1/2 (100), removing the phosphorylation of YAP/TAZ, thus allowing translocation (100, 101). Dephosphorylated YAP and TAZ are then translocated into the nucleus where they bind a family of transcription factors (TEAD family) and induce transcription of genes that promote stem cell activation, proliferation, and increased cell migration (97). Thus, not surprisingly, YAP/TAZ is often upregulated in cancers (102), including breast cancer (103), and serves to help drive cancer progression. Alternatively, knocking down TAZ with shRNA reduced mammosphere formation and decreased overall tumorigenicity in MIV cells (104). In many cancers, such as squamous cell carcinomas, prostate cancer, melanoma, malignant mesothelioma, osteocarcinoma, and neuroblastoma, YAP knockdown impairs growth of their respective cell lines (105).

Importantly, upon ECM stiffening, more YAP/TAZ is found in the nucleus (93), suggesting a potential mechanism for the progression of cancer upon stiffening of the TME. In support of this, multiple studies found that stiffer substrates do cause more YAP/TAZ activation, and this activation is linked to both increases in tumor cell growth and migration and an increased chemoresistance (97, 103). Because YAP/TAZ nuclear localization is sensitive to ECM changes/stiffness, this is another pathway often implicated in cancer transcriptional regulation (106).

### 2.4 Emerin

Emerin is one of the NE proteins able to directly receive signals from the LINC complex through binding SUN proteins at the INM (70, 76, 107, 108), which then ‘decodes’ the signal to affect nuclear structure and/or transcription (**Figure 1**). Studies have shown that applying force on nesprin-1 triggers nuclear stiffening that does not involve chromatin or nuclear actin but requires an intact nuclear lamina and emerin (109). Emerin becomes tyrosine phosphorylated at Y74 and Y95 in response to force, which is required to mediate the nuclear mechanical response to force (109, 110). This includes the activation of mechanosensitive genes, as emerin was shown to activate the mechanosensitive genes IEX-1 and EGR-1 (15, 111).

**Figure 2:**
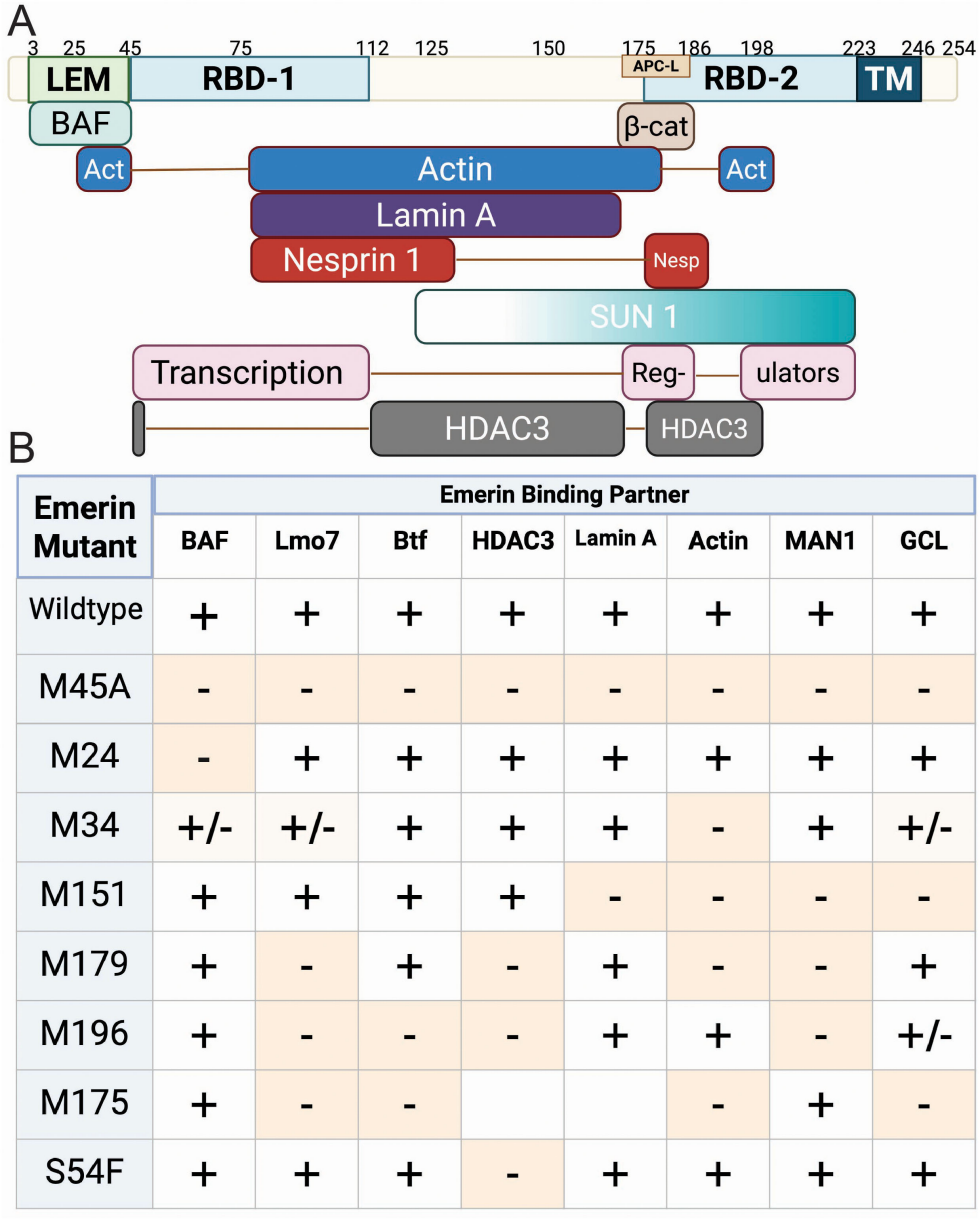
Emerin domain structure. **(A)** Emerin polypeptide with mapped binding domains shown. **(B)** Table of emerin mutants that specifically block binding of emerin to specific partners, where (+) indicates normal binding, (+/-) indicates weak binding, and (-) indicates no binding detected. Naming of scanning alanine mutants is based on previous studies (58, 116, 117, 122, 167). Blank boxes indicate partner was not tested. RBD, RNA-binding domain; TM, transmembrane domain; LEM= LAP2, emerin, MAN1 domain; APC-L, APC-like domain. Created with https://www.BioRender.com. .

Emerin is a ubiquitously expressed INM protein (58, 112). It has a 220-residue nucleoplasmic region, a 23-residue C-terminal transmembrane domain, and an 11-residue luminal domain (**Figure 2A**) (113, 114). Emerin also binds SUN-domain proteins at the INM, linking emerin to the LINC complex and enabling the transmission of mechanical stimuli into the nuclear interior (76). Though exact binding interfaces have not been determined, emerin has been reported to bind directly to residues 209–302 of SUN1, which is named the emerin binding domain (EBD) (76). Outside of the N-terminal ~40-residue LEM domain and the transmembrane domain, emerin is intrinsically disordered (115). The nucleoplasmic domain of emerin binds many different proteins, including transcription regulators, chromatin regulatory proteins, and nucleoskeletal proteins (111). Nucleoskeletal proteins generally bind the central domain of emerin, with lamin A and F-actin binding to residues 64-170 (116, 117). F-actin also requires a few residues from 170-198 (118, 119). Lamin A interacts with emerin at the INM, also contributing to emerin’s INM localization (60). Emerin is a member of the LEM domain proteins, which are known for binding lamins and barrier-to-autointegration factor (BAF), which is involved in tethering chromatin at the INM (**Figure 2A**) (120). LEM-domain proteins regulate gene expression, chromatin architecture, and contribute to nuclear structure (112). Recent reports show emerin, when under stress, may unbind from these partners and self-assemble along its own intrinsically disordered domain and form oligomers that are further stabilized by the LINC complex (121). Emerin also binds a number of other proteins (**Figure 2B**) (111), and these interactions allow emerin to regulate additional cellular functions, such as nuclear architecture through connections with Lamin A or actin, or by altering gene expression through its indirect interaction with transcriptional regulators (117, 122–124) such as MKL1.

Emerin has been directly implicated in MKl1-dependent mechanotransduction (63, 85). Emerin is required for MKL1-mediated activation of SRF-regulated genes in response to substrate stiffening, many of which are cytoskeletal proteins or cell adhesion proteins (63, 109). Disruption of emerin impairs force-induced transcriptional programs and alters nuclear mechanical properties (14, 15, 90), further supporting its role as a mechanosensitive effector downstream of the LINC complex. Ho et al. report lamin A/C and emerin regulate MKL1 signaling through modulating actin polymerization, and disruption of emerin or lamins decreases MKL1-SRF activity (63). In further support of this, Willer and Carroll demonstrated that emerin is required for MKL1-SRF transcription in response to increasing stiffness (85), which positions emerin as an important upstream regulator of MKL1 in response to mechanotransduction.

Emerin may also stimulate nuclear actin polymerization upon receipt of mechanical signals from the LINC complex, as emerin stabilizes F-actin filaments at their pointed end in the INM (125). Given the sensitivity of MKL1 to actin dynamics and cytoskeletal tension, emerin has the potential to influence the MKL1 pathway through its role in actin stabilization (125) and nuclear architecture (126–128). In support of this, previous work also showed that changes in substrate stiffness cause LINC complex-dependent changes in nuclear F-actin and transcription of migratory and invasive genes by MKL1. Importantly, this balance between G-actin and F-actin that regulates MKL1 activity itself is controlled by overall cytoskeletal tension (129). Through its interactions with lamin A (60, 116), chromatin regulators (24, 119), and transcriptional co-regulators such as MKL1 (85, 90), emerin is uniquely positioned to couple mechanical inputs to changes in nuclear and cytoskeletal architecture both directly and through regulation of gene expression.

Similar to MKL1-signaling, emerin is also positioned to contribute to YAP/TAZ regulation, either directly or indirectly, by modulating nuclear mechanics and chromatin reorganization, affecting the transmission of mechanical cue(s) to drive transcriptional programs (54, 93, 109). It will be important to determine whether emerin functions as a mechanosensor in YAP/TAZ-mediated mechanotransduction. In addition to YAP/TAZ signaling, there are many lamin-dependent pathways linked to cancer progression, such as TGF-β, PI3K/AKT, and MAP/ERK signaling (130). However, it is difficult to determine whether such responses are in direct response to lamin loss/disruption or the disruption of the lamina-associated proteins, and thus, more disruption studies are needed.

### 2.5 Nuclear actin

It is worth noting nuclear actin also has functions aside from MKL1 modulation that likely contribute to nuclear architecture and transcription (131). Nuclear actin was shown to interact directly with architectural proteins in the NE (132). Monomeric β-actin was shown to interact with RNA polymerase II (133). In Xenopus oocytes and HeLa cells, nuclear F-actin was shown to alter nuclear shape in the absence of lamin A (134). In addition to MKL1, the balance of G- and F-actin in the nucleus affects activity of other transcription factors, such as PREP2, transcription repressor YY1, and growth factor receptor Erb2, all of which have been shown to be upregulated in cancer subtypes (133, 135–140). These many nuclear actin-related functions likely contribute to the cell’s response to external stimuli, at least in part, due to emerin stabilizing F-actin formation in the nucleus (125) and receiving signals from LINC (70, 76, 107, 108).

### 2.6 Mechanotransduction pathways regulate transcription and nuclear architecture

Mechanosensitive transcription factors are one mechanism by which mechanotransduction regulates gene expression. As stated above, two of the best characterized examples are MKL1 and YAP/TAZ (93, 106). However, mechanotransduction also regulates many other factors and coactivators. The transcription factor SRF not only integrates signals through MKL1, but also through the activation of ELK1, a member of the ternary complex factor (TCF) family activated by MAP/ERK signaling (141, 142). β-catenin-mediated transcription is also influenced by cytoskeletal tension and cell-cell adhesion (143). Activation of NF-κB in response to shear stress and ECM stiffening has been shown to promote expression of genes involved in survival and proliferation (144). These responses do not occur in isolation but likely act cooperatively to regulate nuclear and chromatin architecture and mechanics, further shaping transcriptional outcomes.

In response to increased stress in the extracellular environment, mechanotransduction allows for the cell to adapt by strengthening the cytoskeleton and increasing the expression of genes important for modulating cell contractility and adhesion. For example, activation of RhoA in response to ECM stiffening stimulates ROCK, which promotes actin polymerization and actomyosin contractility (91). ROCK enhances F-actin through phosphorylation of LIM kinase, which inhibits cofilin-mediated actin depolymerization to increase cytoskeletal tension and force transmission (145). In parallel, increased cytoskeletal tension activates mechanosensitive transcriptional regulators such as MKL1 and YAP/TAZ (85, 93), that then activate a number of contractility and adhesion genes, including YAP/TAZ targets *CTGF* and *CYR61* (93, 146) and MKL1 targets *ACTA2, MYH9*, and *TAGLN* (85, 129). Emerin binds directly to nuclear F-actin to stabilize it at the INM (54, 125). Thus, emerin is predicted to regulate nuclear actin dynamics in response to RhoA/ROCK-driven cytoskeletal tension (89, 147).

Forces transmitted to the nucleus not only alter transcription but also directly alter nuclear and subnuclear architecture. The nucleus is dynamic, and the organization of the nuclear lamina, INM proteins, and chromatin contribute to nuclear stiffness (58, 148). Force transmission through the LINC complex allows for mechanical signals to be directly delivered to the NE. In addition to direct mechanical coupling, mechanosensitive pathways such as YAP/TAZ (93, 106) and MKL1 (85, 149) can also influence nuclear architecture by regulating expression of nuclear and cytoskeletal structural genes, such as lamin A/C (*LMNA*) (63) and cytoskeletal genes (*ACTA2, ACTB, VCL, MYH9*) (85, 150). These forces can induce changes in nuclear shape and stiffness and in chromatin organization (56). The nuclear lamina and nuclear F-actin are not the only polymers that contribute to nuclear stiffness, as chromatin compaction and positioning also influence nuclear rigidity, as well as transcriptional accessibility (61, 151). Interestingly, the resultant nuclear architecture changes caused by mechanotransduction are often context-dependent and have opposite effects on nuclear architecture. In some cases, increased cytoskeletal tension is associated with increased lamin A/C organization and nuclear stiffening (152), and in others, nuclei have increased deformability and structural reorganization under mechanical stress (78, 153).

As a downstream effector of the LINC complex, emerin contributes to nuclear architecture regulation through its interactions with lamins, lamin-associated proteins, and chromatin-associated proteins (24, 58, 126). Mechanical force directly applied to nesprins induces emerin phosphorylation at Y74 and Y95, which is required for nuclear stiffening and the adaptive nuclear responses to force (109, 110). Beyond nuclear architecture, emerin also interacts with lamin A/C and chromatin-binding proteins such as HDAC3, BAF, G9a, and EZH2 (111), which promotes the organization of repressive chromatin at the nuclear periphery (24, 154, 155). Regions of repressive chromatin associated with the NE are called Lamina Associated Domains (LADs) and consist of both facultative and constitutive heterochromatin (24, 156, 157). Emerin is required for the localization of repressive chromatin to the NE (119, 158), which is mediated by its association with HDAC3 (119). Further, emerin deficient cells have more open chromatin organization (159–161) as well as decreased repressive methylation marks, H3K9me2/3 and H3K27me3, and increased H4K5ac, the substrate of HDAC3 (119). Emerin interacts with histone methyltransferases EZH2 and G9a, which methylate H3K27 and H3K9, respectively, presumably to help maintain stable LADs (24, 61). Interestingly, knocking down emerin also disrupted repressive chromatin localization in retinal pigment epithelial cells, in which repressed chromatin that is normally in the interior of these cells, was now moved to the nuclear periphery (162). Therefore, mechanotransduction through emerin may also affect nuclear integrity by regulating chromatin organization.

## 3 Emerin, mechanotransduction, and disease

### 3.1 The nucleus, emerin, and breast cancer

Because mechanotransduction regulates nuclear and cytoskeletal architecture and motility, disruption of mechanosensitive pathways has major impacts on disease progression. Emerin and the LINC complex have independently been linked to regulation of mechanosensitive genes (15, 63, 66, 84, 109, 163). Not surprisingly, these critical processes are often found to be disrupted in the context of diseases such as cancers, where the TME plays a key role in cancer transformation and metastasis.

The nucleus, which is 10-20 μm in diameter (52), serves as a major physical barrier to metastasis in cancer, as cancer cells must be able to squeeze through small slits in the endothelial cells lining the vasculature (~1-2 μm in diameter) (164). Therefore, cells, and the nucleus in particular, must go through massive physical changes to squeeze through these small gaps to initiate the process of metastasis. Thus, this nuclear softening was recognized as a “hallmark of cancer” (58). These nuclear structural changes are shown to be governed in part by emerin, whose levels are associated with patient survival (126). Not coincidentally, pathologists use nuclear morphology as a visual criterion for cancer diagnosis in both breast and thyroid cancers (127, 165, 166).

Triple-negative breast cancer (TNBC) cells, which are highly invasive and metastatic, also have less emerin protein expression compared to less invasive and non-invasive cell lines (126, 128). This decreased emerin protein expression is accompanied by decreased nuclear size, aberrant nuclear structure, and increased migration through micron-sized pores (126, 128). Expression of GFP-tagged emerin in these cells rescued both the nuclear structural defects and the impeded cell migration defects (58, 126), suggesting emerin blocks metastatic progression. Because of emerin’s interaction with many other partners, a library of alanine-substitution mutants were created that disrupt binding to one or multiple partners (**Figure 2B**) (58, 116, 117, 122, 167). To determine which of emerin’s many functions are involved in this process, emerin mutants that disrupted nucleoskeletal and transcriptional binding partners were tested for altering nuclear area, circularity, volume, and cell migration in TNBC cells. It was found that emerin mutants that did not bind nucleoskeletal partners were unable to rescue nuclear size and cell migration to non-cancerous levels (126). In orthotopic mouse models of breast cancer, wildtype emerin expression in MDA-231 cells decreased primary tumor growth. Most relevant to the migratory function of breast cancer cells, wildtype emerin blocked lung metastasis (126). Emerin mutants unable to bind the nucleoskeletal partners lamin A and actin were unable to block tumor growth or metastasis (126). This effect was dependent on emerin’s ability to bind to lamin A and actin, as wildtype emerin and emerin mutants unable to bind transcription factors eliminated lung metastasis, demonstrating that emerin’s function in metastatic spread is likely dependent on its role in regulating the nucleoskeleton (126).

In breast cancer patients, SUN1/2 and nesprins are downregulated (168), and disruption of LINC complex signaling reduces nuclear F-actin (84), decreases nuclear size (169), alters MKL1 transcription (149), and modulates EMT (168). As emerin binds directly to SUN1 and is a major effector of LINC complex signaling (54, 64, 108, 109, 147). it is possible that the loss of the emerin-LINC complex interaction is a driver for enabling metastatic transformation (109). In support of this, emerin-null cells have more malleable nuclei compared to their emerin-expressing counterparts when measured via micropipette aspiration, where specific pressures were applied to cells and imaging tracked the geometry of the nucleus over time in response to varying pressures (14, 15). Emerin protein expression is also reduced in patients with more advanced cancer, and its expression is negatively correlated with cancer prognosis (126, 170). Because of emerin’s role in mechanotransduction and its reduced protein expression in breast cancer (126), data supports that emerin acts as a critical mechanosensor, altering nuclear structure in response to increasing TME stiffness, to help drive breast cancer metastasis (**Figure 1**).

### 3.2 Emerin, mechanotransduction and other cancers

Emerin is not only implicated in breast cancer. Recent studies report that emerin is also reduced in prostate cancer, and this is accompanied by decreased nuclear integrity, increased cell migration and invasion, and increased metastasis *in vitro* and *in vivo (*171, 172*).* Emerin is also important for reassembling the nucleus after mitosis (173, 174). Thus, using emerin as a potential target for cancer therapy may provide therapeutic benefit because it has the ability to affect both tumor growth and metastasis by modulating nuclear structure (14, 15, 58, 126, 127), and restoring emerin in such cells has the potential to prevent nuclear structural deficiencies that allow for easier migration and invasion. We predict loss of INM-localized emerin potentiates cancer progression through its inability to respond to mechanical signals transmitted through the LINC complex to alter nucleoskeleton organization and/or transcription.

LINC complex dysregulation is implicated in cancer progression and metastasis (25–29). LINC complex disruption promotes genomic instability (175, 176) through its influence on chromatin architecture, likely through emerin since emerin regulates chromatin architecture (24, 167). Upon disruption of the LINC complex, chromatin becomes less compacted (177), thereby increasing transcriptional accessibility (178). Changes in this chromatin organization and nuclear architecture alter the proximity of chromosomes to one another, potentially increasing the likelihood of oncogenic translocations (179, 180). In breast cancer, focal amplifications from intrachromosomal translocations are known contributors to the amplification of well-known oncogenes such as HER2 and cyclin D1, as well as additional loci that remain under investigation (181). These findings emphasize the importance of nuclear-cytoskeletal coupling and suggest emerin and LINC dysregulation can drive or contribute to cancer progression.

### 3.3 Emerin, mechanotransduction and other diseases

Mutations in *EMD* cause Emery-Dreifuss muscular dystrophy (EDMD), which alters muscle function and integrity in patients and occurs in approximately 1–2 per 100,000 births (182–185). Most EDMD1-causing *EMD* mutations are nonsense mutations (186, 187). Since emerin is a protein that receives signals directly from the LINC complex to dynamically alter cytoskeletal and nucleoskeletal architecture, and gene expression profiles (64, 70, 107), it is predicted that reduced emerin attenuates activation of mechanosensitive genes involved in muscle maintenance and repair to cause the progressive skeletal muscle weakness and degeneration (188). Additionally, as emerin interacts with repressive chromatin machinery (24), emerin loss is predicted to alter the myogenic differentiation gene expression program. Dysregulated mechanotransduction is also implicated in diseases such as osteoporosis and osteoarthritis, where a breakdown in mechanotransduction impairs osteogenesis, causing progressive bone loss over time (55). In polycystic kidney diseases, kidney tubules fail to sense mechanical signals properly, leading to abnormal cell growth and cyst formation (189). Genetic mutations in the mechanosensitive channels of the inner ear can contribute to sensorineural hearing loss (190). As illustrated through these diseases, successful mechanotransduction is required for proper tissue and organ function.

## 4 Discussion: limitations and new technology

Experimental approaches for measuring stiffness have been optimized to provide reliable, quantitative measurements of both biological specimens and engineered matrices or beads. These measurements are particularly important because tissue stiffness is dynamic, undergoing both reversible and irreversible changes throughout development, aging, and disease (191–198). By understanding tissue stiffness in healthy patients, the detection of abnormal growth and the changes in the surrounding microenvironment will become easier to identify (30).

Recent innovations in measuring stiffness have allowed for accurate stiffness measurements in real-time. Approaches like Atomic Force Microscopy (AFM) and elastography-based techniques have been used to quantify stiffness in both clinical and experimental settings (199) due to their ability to measure matrices, tissues, and cell membranes at a nanoscale level (30, 200). Optical tweezers are used experimentally because they measure stiffness at the cellular level (201–203). Micromanipulation force measurements have also been acquired on isolated nuclei using modified micropipette aspiration, whereby one pipette pulls on the nucleus while the other one detects the force on the nucleus (203, 204). In clinical settings, Magnetic Resonance Elastography (MRE) is often used to measure stiffness of tissues deep within the body (205) and shear wave elastography (SWE) can be used for tissues closer to the skin, such as in the breast (206).

Despite significant advancements in identifying mechanotransduction pathways, limitations remain in studying how mechanical signals are transmitted and interpreted at the molecular level. One challenge is determining which specific protein interactions within a complex or series of complexes are responsible for transmitting and responding to these forces from the ECM. For example, the LINC complex is comprised of many proteins, and it is difficult to ascertain which specific protein interactions are responsible for a specific response. Because these proteins form a continuous link, disruption of one protein will likely indirectly affect the entire system or even affect unrelated pathways.

The use of molecular biosensors combined with microscopy has enabled imaging of the LINC complex in response to mechanical signaling and the mechanosensitive processes in real-time. Recently, Förster (or Fluorescence) resonance energy transfer (FRET) biosensors have been developed to overcome prior imaging limitations. For example, the Conway lab successfully developed FRET-based force sensors such as the nesprin-2G biosensor to study actomyosin-dependent tension (207) and FRET-based nanobodies to study lamin strain (59). Similarly, the King lab used FRET-based tension sensors to investigate force transmission by nesprins across the LINC complex (57). These approaches will allow investigators to determine *how* forces are transmitted to the nucleus and what components of LINC, emerin, and related proteins are *sufficient* to respond appropriately to changes in the TME.

An additional concern is whether isolated nuclei or 2-D cell culture is an accurate representation of physiological conditions. This is exemplified by the observed differences between 2-D and 3-D systems. For example, 2-D cell culture of squamous carcinoma cells showed reduced chemoresistance (166) and less apoptosis resistance (208) than the cells grown in 3-D environments (209, 210). Additionally, cell morphology and cytoskeletal organization differ in both systems, where 2-D environments lead to more cell spreading than in 3-D environments, which adopt more physiologically-relevant shapes (208, 211, 212).

Experimental approaches on isolated nuclei are particularly useful for specifically targeting the nuclear mechanisms involved in mechanotransduction because it removes the contributions of the cytoplasm and cytoskeleton. However, it is yet to be determined whether mechanisms defined using isolated nuclei are translatable to live cells. This is an important consideration because nuclear structure and function is influenced by cytoskeletal forces and extracellular cues that may have different inputs (i.e., LINC and MKL1) and act cooperatively or antagonistically in cells. These limitations emphasize the importance of developing experimental approaches that integrate high-resolution with physiologically-relevant systems in dynamic cellular processes, such as mechanotransduction.

## 5 Conclusion

Mechanotransduction enables cells to integrate mechanical inputs from their environment with biochemical signaling pathways to regulate cellular behavior. Due to the importance of the TME in cancer progression, the study of mechanotransduction and mechanobiology in cancer has recently exploded, with over 23,000 publications linking “cancer” and “mechanotransduction” since 2010 alone. This increase in activity over the last decade highlights the importance of understanding how cellular environments affect cell fate and function.

The LINC complex is a central conduit for cells to sense changes in physical stimuli from the ECM or TME at the plasma membrane and transmit this stimulus to the nucleus. It has become increasingly clear that cancers exploit this mechanotransduction pathway to increase their proliferation, cell migration, and cell invasion through modulating both gene expression and nuclear architecture. Alterations in LINC and its downstream effectors, such as emerin, are observed at the protein level in a number of cancers (14, 15, 70, 126, 168), concomitant with changes in nuclear structure and the appearance of tumorigenic characteristics (126). These findings support emerin as a key downstream effector linking altered LINC function to nuclear softening and increased cell migration and invasion seen upon tumor stiffening.

Many fundamental questions remain unresolved regarding nuclear mechanotransduction and emerin function. For example, the mechanism for *how* signals from the TME are transduced through the LINC complex to the nucleus and what role emerin specifically has in this process is not clear. As emerin and the LINC complex are both linked to the regulation of mechanosensitive genes (84), it is not yet known if emerin responds to LINC complex signaling by eliciting a direct or indirect transcriptional response. Although emerin is downregulated in several cancers, including TNBC (58, 126), how emerin contributes to mechanotransduction during transformation remains to be defined.

Across biological scales, an important challenge moving forward will be to understand how pathways differ in different contexts, such as in 2-D and 3-D environments and between cells, tissue, and entire organisms. Addressing these questions requires continued advancements in technologies focused on assessing mechanotransduction pathways at the molecular level in a physiologically relevant way.

Collectively, current data supports emerin as not simply a structural component of the LINC complex but as a *mechanosensor* that integrates mechanical ECM signals with transcriptional outputs. It is yet to be determined *how* these forces are integrated, which is especially important in the context of cancer. As our understanding of mechanotransduction increases, it may reveal emerin as a promising target for cancer therapeutics.
